# UV C Light from a Light-Emitting Diode at 275 Nanometers Shortens Wound Healing Time in Bacterium- and Fungus-Infected Skin in Mice

**DOI:** 10.1128/spectrum.03424-22

**Published:** 2022-12-01

**Authors:** Chenghua Song, Ruichao Wen, Jiaxuan Zhou, Xiaoyan Zeng, Zi Kou, Yufeng Li, Feng Yun, Rongqian Wu

**Affiliations:** a National Local Joint Engineering Research Center for Precision Surgery & Regenerative Medicine, Shaanxi Provincial Center for Regenerative Medicine and Surgical Engineering, The First Affiliated Hospital of Xi’an Jiaotong University, Xi’an, China; b Solid-State Lighting Engineering Research Center, Xi’an Jiaotong University, Xi’an, China; c Department of Laboratory Medicine, The First Affiliated Hospital of Xi’an Jiaotong University, Xi’an, China; d Oujiang Laboratory (Zhejiang Lab for Regenerative Medicine, Vision and Brain Health), Wenzhou Institute, University of Chinese Academy of Sciences, Wenzhou, China; Columbia University

**Keywords:** skin and soft tissue infections, UVC-LED, 275 nm, MRSA, *Candida albicans*

## Abstract

Due to the changes in pathogenic species and the absence of research on topical skin antibiotics, the therapy of skin and soft tissue infections (SSTIs) is facing more and more severe challenges. It is particularly urgent to look for alternative therapies without induction of drug resistance. UV C (UVC) light within the range of 200 to 280 nm is one of the most common techniques used to kill and/or inactivate pathogenic microorganisms. However, the traditional most commonly used wavelength of 254 nm irradiated from a low-pressure mercury lamp is hazardous to human health, being both carcinogenic and damaging to eye tissues, which limits its applications *in vivo*. This research aimed to investigate the antimicrobial properties and influence of 275-nm UVC light from a light-emitting diode (UVC-LED light) on wound healing time. Five bacteria, three fungi, and scalded-mouse models combined with SSTIs were used to evaluate the antimicrobial effect *in vitro* and *in vivo*. 275-nm UVC-LED light inactivated both bacteria and fungi with a very short irradiation time *in vitro* and induced neither DNA damage nor epidermal lesions in the mice’s skin. Furthermore, in mouse models of SSTIs induced by either methicillin-resistant Staphylococcus aureus (MRSA) or Candida albicans, the 275-nm UVC-LED light showed significant antimicrobial effects and shortened the wound healing time compared with that in the no-irradiation group. UVC-LED light at 275 nm has the potential to be a new form of physical therapy for SSTIs.

**IMPORTANCE** As a common clinical problem, the therapy of SSTIs is facing growing challenges due to an increase in the number of drug-resistant bacteria and fungi. UV C (UVC) light sterilization has been widely used in all aspects of daily life, but there are very few reports about *in vivo* therapy using UVC light. It is well known that prolonged exposure to UVC light increases the possibility of skin cancer. In addition, it is also very harmful for eyes. UV irradiation with 254-nm UVC light can cause corneal damage, like thinning of the corneal epithelial layer, superficial punctate keratitis, corneal erosion, etc. In this study, we focused on looking for a more accessible light source and safer UVC wavelength, and 275-nm UVC LED light was chosen. We investigated its applicability for SSTIs therapy with relative skin safety and expected that it could be used as a new physical therapy method for SSTIs.

## INTRODUCTION

Skin and soft tissue infections (SSTIs) are a common clinical problem, associated with surgical incisions, burns, pressure sores, diabetic foot infections, etc., ranging from mild superficial forms to severe life-threatening infections (1, 2). SSTIs affect the healing of the wound, resulting in difficult repair or irreparable wounds (3, 4). More seriously, SSTIs can induce systemic infection, sepsis, amputation, and even death (5–7). Therefore, the prevention and treatment of infection have always been an important link in the rehabilitation of patients with SSTIs (8), and antimicrobial treatment is particularly important (9).

The management of SSTIs is becoming an increasing challenge. Antibiotics are the first choice for the clinical treatment of SSTIs (10, 11). However, research on topical skin antibiotics is sorely lacking (12, 13), the research cost of new antibiotics is relatively high, and the development cycle is much longer than the breeding speed of drug-resistant microbes (10, 14). Few novel skin antibiotics have been available in recent years (15–18). In addition, the focus of anti-SSTIs in the clinic for a long period has been on fighting bacteria; fungal infections have been somewhat neglected. Studies have shown that the prevalence of superficial mycosis worldwide is as high as 20% to ~25%, indicating that fungi have partially replaced bacteria as the main pathogens (19–21). Untreated aggravation of deep SSTIs by Candida albicans can cause rapid necrosis of tissues and organs or spread into the blood, resulting in fungal septicemia and sepsis and even threatening the patient’s life (22). The situation of fungal infections is increasingly serious. Unfortunately, most existing antimicrobials tend to work against only bacteria or fungi, not both. Therefore, it is particularly urgent to look for alternative therapies for SSTI treatment that are effective against both bacteria and fungi.

UV C (UVC) light within the range of 200 to 280 nm is in widespread use for disinfection of water (23–25), food processing (26), and air disinfection of hospitals, laboratories, and daily life (27). It is one of the most common techniques for inactivating pathogenic microorganisms (28). There has been significant research evaluating the use of UVC wavelengths to inactivate organisms in other media and in the human health field, but actual widespread use in those other fields has not yet been realized due to a variety of concerns, including exposure of human skin and eyes to UVC wavelengths (29). In China and some European countries, there are some reports about the combination of UV light and red light in the treatment of diabetic foot ulcers in the clinic (30). Some of the associated devices have been licensed as medical devices and are in clinical use. For example, the shortwave UV therapy apparatus (wavelength, 253.7 nm) manufactured by Beijing JD Healthcare Equipment. Co., Ltd., has been used in a variety of superficial infectious diseases, and there are similar devices in development. Therefore, we believe that sterilizing devices based on UV light still have a certain clinical application prospect. Bacteria and fungi are unlikely to develop resistance to UVC light used as a form of physical sterilization, and UVC light has been proven to inactivate both drug-sensitive and multidrug-resistant bacteria, fungi, and even viruses (human immunodeficiency virus and severe acute respiratory syndrome coronavirus 2, etc.) (31–33). However, the traditional most commonly used wavelength of 254 nm irradiated from low-pressure mercury lamps is hazardous to human health, being both carcinogenic and damaging to eye tissues, which limits its applications *in vivo* (34). The rapid development of new light sources in recent years has prompted more research on the antimicrobial effect and safety of different UVC wavelengths. Because it induces neither DNA damage nor epidermal lesions in mouse skin and cornea (35), most *in vivo* studies adopt the 222-nm wavelength (28, 36–39). There is growing interest in the use of filtered UVC light at 222 nm, but few studies have reported successful construction of a 222-nm UVC-LED light source with high quality, and the light source is unavailable in many counties. It has not yet attained widespread use. The emergence of the light-emitting diode (LED) light source provides a new option for UVC sterilization and realizes the continuously tunable wavelength (40). There has been limited research on the 265-nm wavelength (34, 41–45) and 280-nm wavelength (46–49) for treatment recently. However, no literature reported the *in vivo* application and safety of 275-nm UVC light in antimicrobial therapy (32). Due to the strong characteristic absorption peak at 270 to 280 nm of tryptophan and tyrosine, the 270- to 280-nm wavelength is considered to inactivate microbes mainly by destroying the protein structure of the microbes and interfering with the function of the proteins (50). Therefore, the 270- to 280-nm wavelength induces less damage to the nucleic acid (absorption wavelength ranges of nucleic acids, 250 to 265 nm). In addition, the 275-nm UVC-LED light source has been made commercially available, and a high-quality light source can be easily accessed from the market. This makes the design of a personalized light source based on this wavelength relatively easy and feasible. Researchers can design and manufacture point light sources, line light sources, surface light sources, and other kinds of light source matrices of different shapes and sizes using 275-nm UVC-LED chips according to their own application requirements.

**FIG 1:**
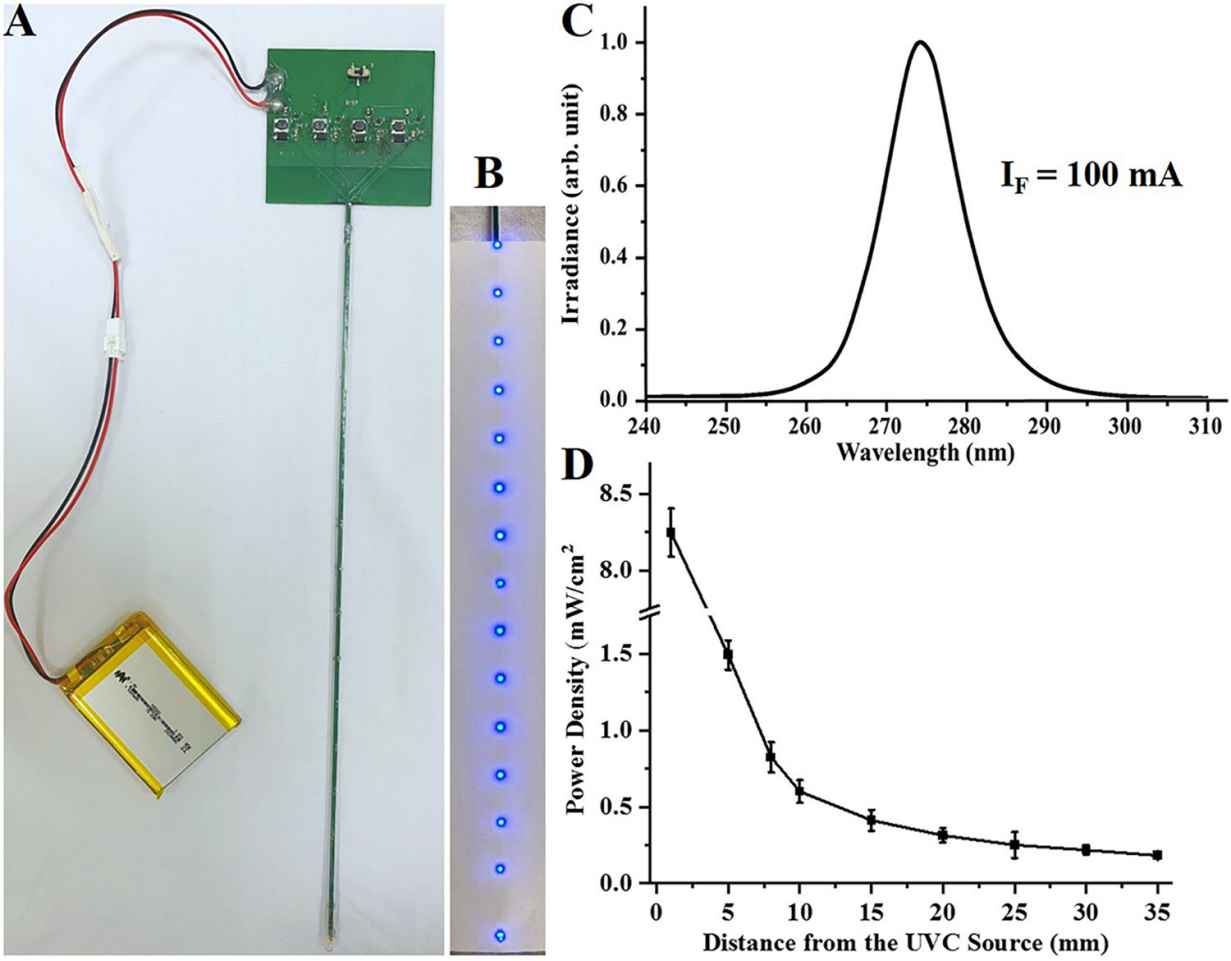
Characteristics of the designed 275-nm UVC-LED lamp tube. (A and B) The appearance of the designed 275-nm UVC-LED lamp tube. (C) Irradiance spectra emitted from the 275-nm UVC-LED lamp tube. (D) Irradiance at different distances from the 275-nm UVC-LED light source.

In this study, as illustrated in Fig. 1, a UVC-LED lamp tube with a central irradiation wavelength of 275 nm (275 ± 10 nm) was self-designed and assembled, and the antimicrobial effect and safety of the 275-nm UVC light were systematically investigated. Meanwhile, the therapeutic effect of the 275-nm UVC-LED light on scalded-mouse models combined with SSTIs by methicillin-resistant Staphylococcus aureus (MRSA) or Candida albicans was studied to determine its applicability. The results demonstrated that the 275-nm UVC-LED light showed a broad-spectrum antimicrobial effect for both bacteria and fungi, inducing negligible damage to mice skin. It can also shorten the wound healing time for SSTIs induced either by MRSA or by Candida albicans *in vivo* and has shown great potential, thus making it a promising novel physical therapy for SSTIs and other types of epidermal infections.

## RESULTS

### UVC-LED light at 275 nm potently inactivates a broad range of both bacteria and fungi.

**FIG 2:**
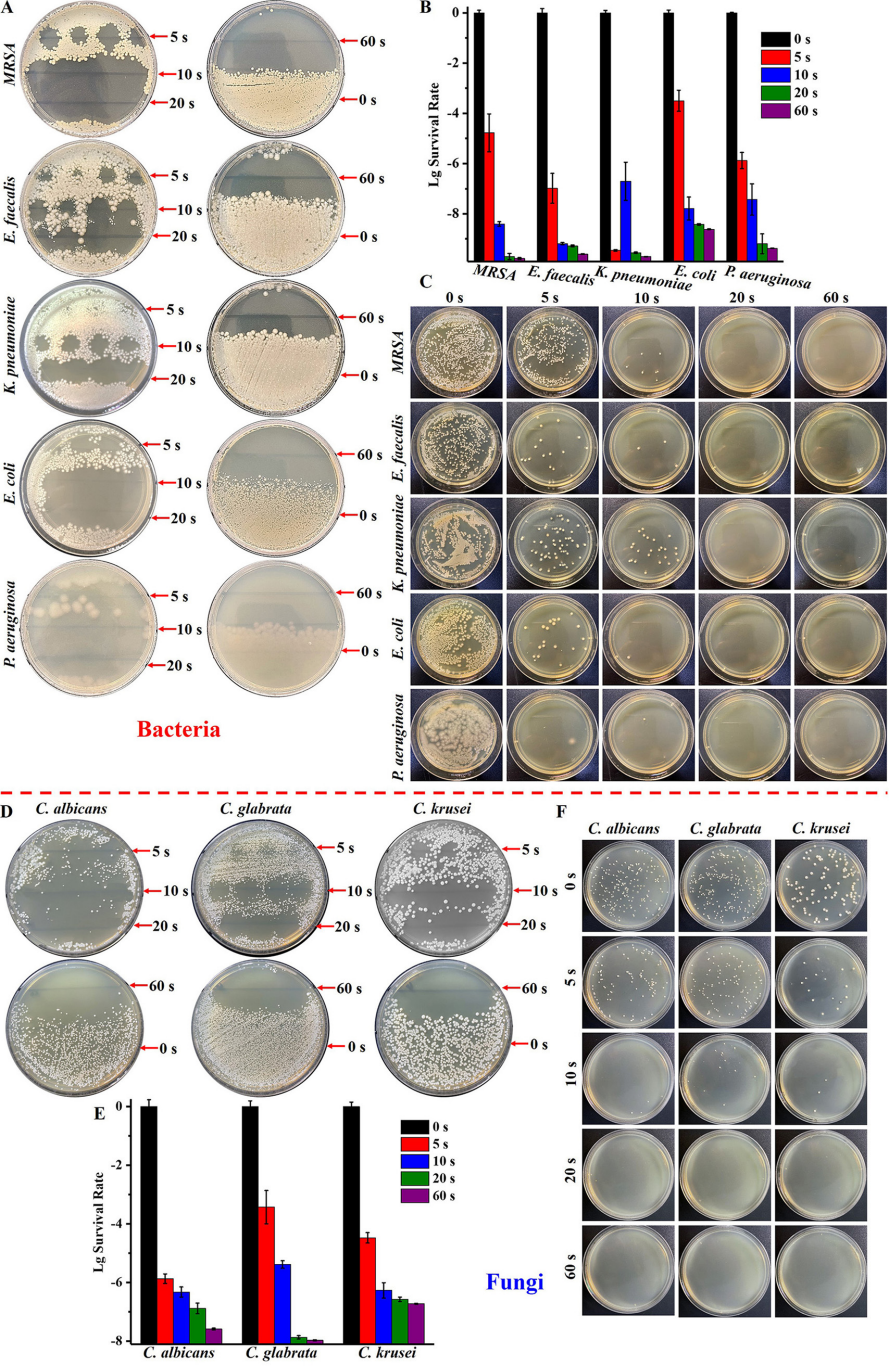
Broad-spectrum antimicrobial effect of the 275-nm UVC-LED light *in vitro* (*n* = 3). (A and D) Growth of the colonies of strains directly irradiated by the 275-nm UV-LED light for 0 s, 5 s (7.5 mJ/cm^2^), 10 s (15 mJ/cm^2^), 20 s (30 mJ/cm^2^), or 60 s (90 mJ/cm^2^) in culture dishes. The distance between the lamp tube and agar surface was ~5 mm, and irradiance was ~1.5 mW/cm^2^ (dose = irradiance × irradiation time). (B and E) Survival rate of strains after irradiation by the 275-nm UV-LED light for 0 s, 5 s (25 mJ/cm^2^), 10 s (50 mJ/cm^2^), 20 s (100 mJ/cm^2^), or 60 s (300 mJ/cm^2^) in saline solution in a self-designed 3D printed culture plate. Strains were transferred into 96-well culture plates after irradiation and cultured for 12 h. The distance between the lamp tube and liquid surface was ~3 mm, and irradiance was ~5 mW/cm^2^. (C and F) Growth of the colonies of strains after the same operations as for panels B and E.

As shown in Fig. 2A and D and in Fig. S1 in the supplemental material, bacteria or fungi inoculated in culture dishes were directly irradiated by the 275-nm UVC-LED light with an irradiance of ~1.5 mW/cm^2^. With the increase of irradiation time (from 5 s [7.5 mJ/cm^2^] to 20 s [30 mJ/cm^2^]), the growth inhibition area changed from small cycles to a big area. Half of the area of the culture dish of microbes was inhibited under 60 s (90 mJ/cm^2^) of irradiation. In contrast, the microbes that were not irradiated grew well. Even after only 10 s (15 mJ/cm^2^) of irradiation, obvious aseptic zones were formed in the culture dishes of MRSA, Escherichia coli, Pseudomonas aeruginosa, and C. albicans. No colony growth was observed for any of the eight microbial strains after 60 s of irradiation. The distinguished antimicrobial effect of the 275-nm UVC-LED light was further confirmed when the eight strains were dispersed into liquid and irradiated (Fig. S2). Figure 2B and E show the survival rate of strains after irradiation by the 275-nm UVC-LED light in 30 μL of saline solution (thickness, ~0.7 mm) with an irradiance of ~5 mW/cm^2^. Irradiation with the 275-nm UVC-LED light for 5 s (25 mJ/cm^2^) reduced the survival of P. aeruginosa by more than 5 orders of magnitude in the saline solution, and irradiation for 20 s (100 mJ/cm^2^) inactivated almost all the E. coli organisms (more than 8 orders of magnitude) in the saline solution. After 60 s (300 mJ/cm^2^) of irradiation, all five bacteria were below the detection limit (Fig. 2B). The antimicrobial effect of the 275-nm UVC-LED light in inactivating fungi was even more impressive with more than 6 orders of magnitude C. albicans and Candida krusei and more than 5 orders of magnitude of Candida glabrata being inhibited after only 10 s (50 mJ/cm^2^) of irradiation. After 60 s (300 mJ/cm^2^) of irradiation, all three fungi were also below the detection limit (Fig. 2E). Similar results were obtained after the strains were transferred into culture dishes after irradiation in saline solution and monitoring of the growth of colonies. No bacterial or fungal growth was observed after irradiation with the 275-nm UVC-LED light for 60 s with a 300-mJ/cm^2^ dose (Fig. 2C and F).

### UVC-LED light at 275 nm shortens the wound healing time in mice with SSTIs induced by MRSA.

**FIG 3:**
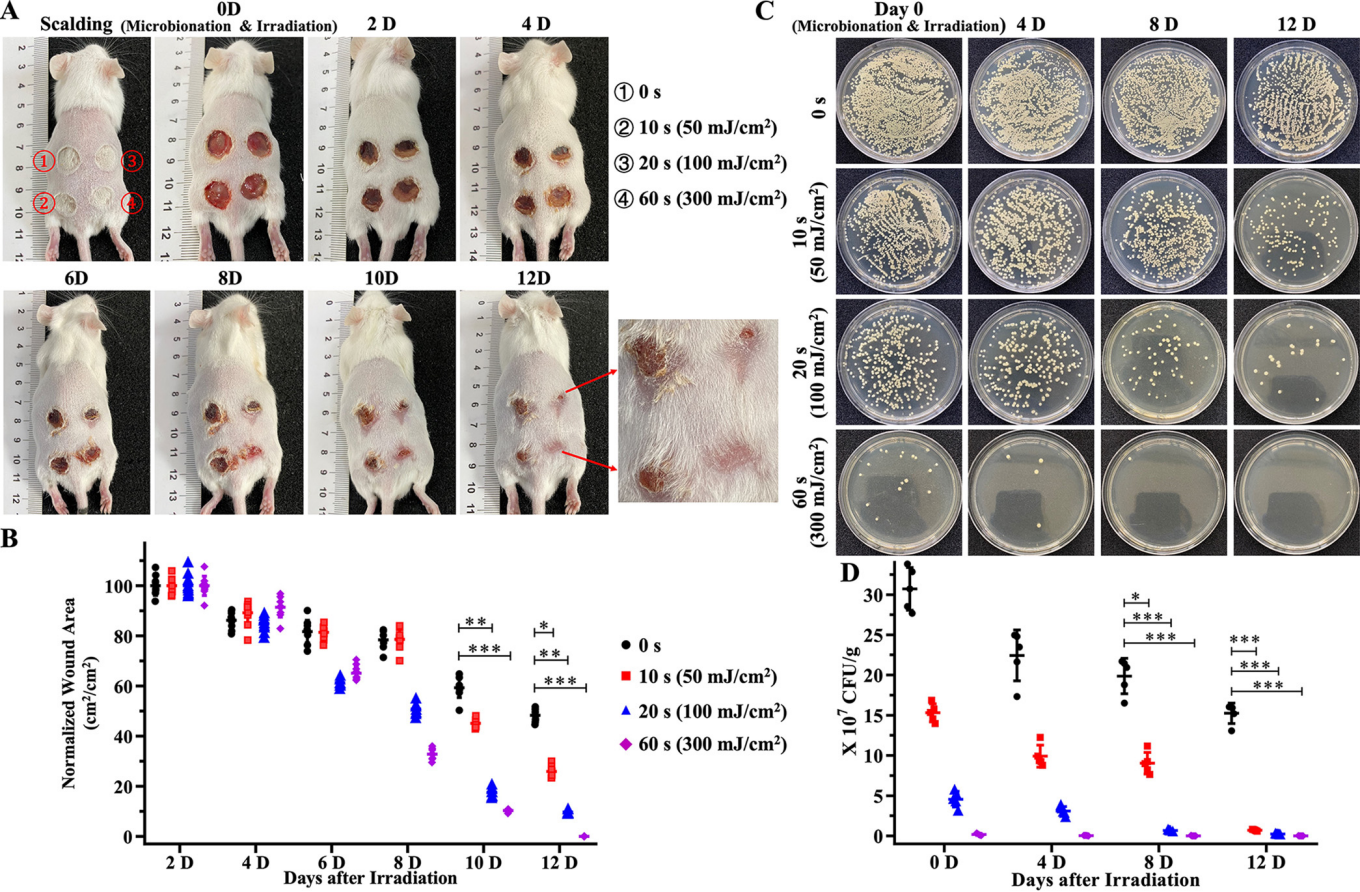
Bactericidal effect and influence on the wound healing time of the 275-nm UVC-LED light on MRSA inoculated onto the scalded dorsal skin of mice. (A) The infected dorsal skin of the mice was treated with the 275-nm UVC-LED light for 0, 10 s (50 mJ/cm^2^), 20 s (100 mJ/cm^2^), or 60 s (300 mJ/cm^2^) with a power density of ~5 mW/cm^2^. (B) Quantitative analyses of wound area by ImageJ software (*n* = 10). (C and D) Time course bacterial counts in the MRSA-infected wounds after treatment with the 275-nm UVC-LED light for different times. Skin tissues of different groups were sampled on days 0, 4, 8, and 12. Tissue suspensions were diluted with sterile saline solution and plated in LBA culture dishes (*n* = 5). Images were taken 18 to 24 h after inoculation. *, *P* < 0.05; **, *P* < 0.001; ***, *P* < 0.001.

As shown in Fig. 3A and B, the wounds were irradiated with the 275-nm UVC-LED light for 0 s, 10 s (50 mJ/cm^2^), 20 s (100 mJ/cm^2^), or 60 s (300 mJ/cm^2^). There was no significant difference between the four groups in the first 4 days after irradiation by the 275-nm UVC-LED light. On day 8, the scab fell off first in the 60-s irradiation group and the scab area was significantly reduced (34.66% ± 6.20% unhealed). In contrast, there were still edematous areas around the wounds and scabs tightly adhered to the tissue in the 20-s (55.48% ± 7.91% unhealed), 10-s (78.73% ± 4.13% unhealed), and 0-s (77.75% ± 3.23% unhealed) irradiation groups. The wounds in the 60-s irradiation group had completely disappeared on day 12, whereas 10.26% ± 4.69% of unhealed wounds remained observable in the 20-s irradiation group. In contrast, the healing of wounds was still very poor in the nonirradiation (48.39% ± 2.60% unhealed) and 10-s (26.13% ± 2.12% unhealed) irradiation groups. The recovery period was significantly different between 275-nm UVC-LED light-treated and nontreated groups. These results suggest that irradiation by the 275-nm UVC-LED light may shorten the wound healing time of mice against SSTIs induced by MRSA. In addition, the colonizing MRSA organisms on the skin and in subcutaneous tissues were quantified. As shown in Fig. 3C and D, the MRSA strains inoculated onto the scalded tissues were inactivated directly by the 275-nm UVC-LED light. In the 10-s and 20-s irradiation groups, the growth of MRSA was delayed in the previous few days, indicating that the 275-nm UVC-LED light did harm the bacteria in the infected skin tissue. In the 60-s irradiation group, there were very few MRSA colonies in the skin tissues. With the extension of irradiation time, the amount of MRSA colonies left in the wound tissue decreased, and the load of MRSA in the wound skin tissue was positively correlated with the wound healing speed, suggesting that the 275-nm UVC-LED light did shorten the wound healing time of SSTIs by inactivating the pathogenic bacteria.

**FIG 4:**
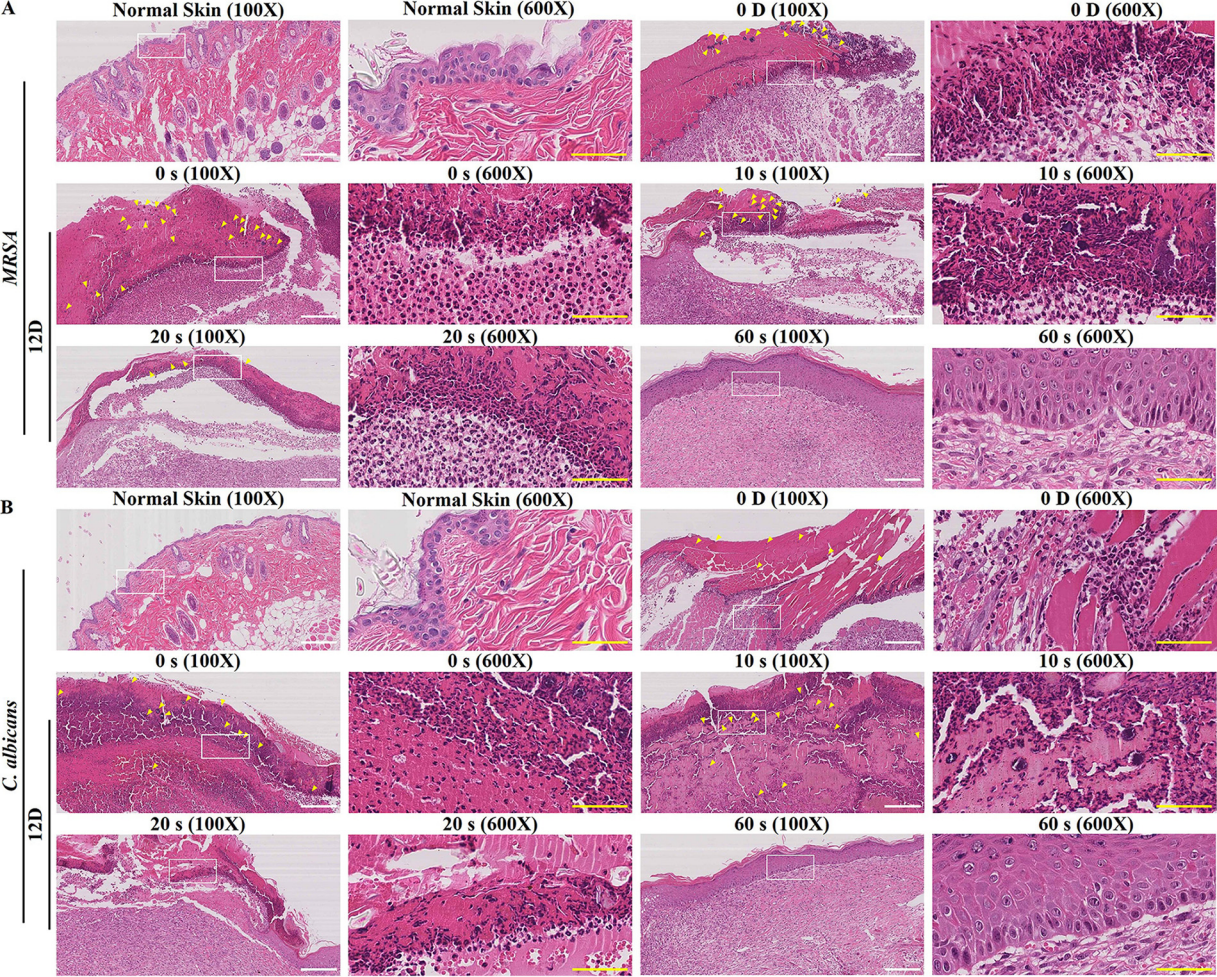
Histology analyses of skin samples of the scalded mice. (A) Mice were infected with MRSA. (B) Mice were infected with C. albicans. The skin wound was treated with the 275-nm UVC-LED light for 0, 10 s (50 mJ/cm^2^), 20 s (100 mJ/cm^2^), or 60 s (300 mJ/cm^2^), with an irradiance of ~5 mW/cm^2^. Skin samples were harvested on day 0 and day 12 after irradiation. White scale bar, 200 μm; yellow scale bar, 50 μm.

As shown in Fig. 4A, compared with normal skin tissues, large amounts of big MRSA clumps were observed in the infected skin tissues on day 0. The clumps were concentrated mainly on the surface of bare muscle tissues, which were accessible for penetration by the 275-nm UVC-LED. There were almost no observable clumps in the deep muscle tissues. Meanwhile, large amounts of fragments of pus cells, inflammatory cell infiltration (mainly neutrophils), and necrotic tissues were also visible throughout the slice. On day 12 after treatment, no MRSA clumps or pus cell fragments were observed in the 60-s irradiation group. Instead, significant neovascularization and fibroblasts were observed in the subcutaneous tissue, indicating the good healing of wounds. In contrast, many smaller MRSA clumps were still observable in the nonirradiated group, and most of these MRSA clumps had penetrated deeply into the muscle tissues, suggesting the importance of treatment at an early stage. Moreover, there was still a massive neutrophil infiltration and a small number of necrotic tissues. The growth and healing conditions of wounds were not good. In addition, the 10-s and 20-s irradiation groups showed a middle therapeutic effect with a small number of tiny MRSA clumps.

### UVC-LED light at 275 nm shortens the wound healing time in mice with SSTIs induced by C. albicans.

**FIG 5:**
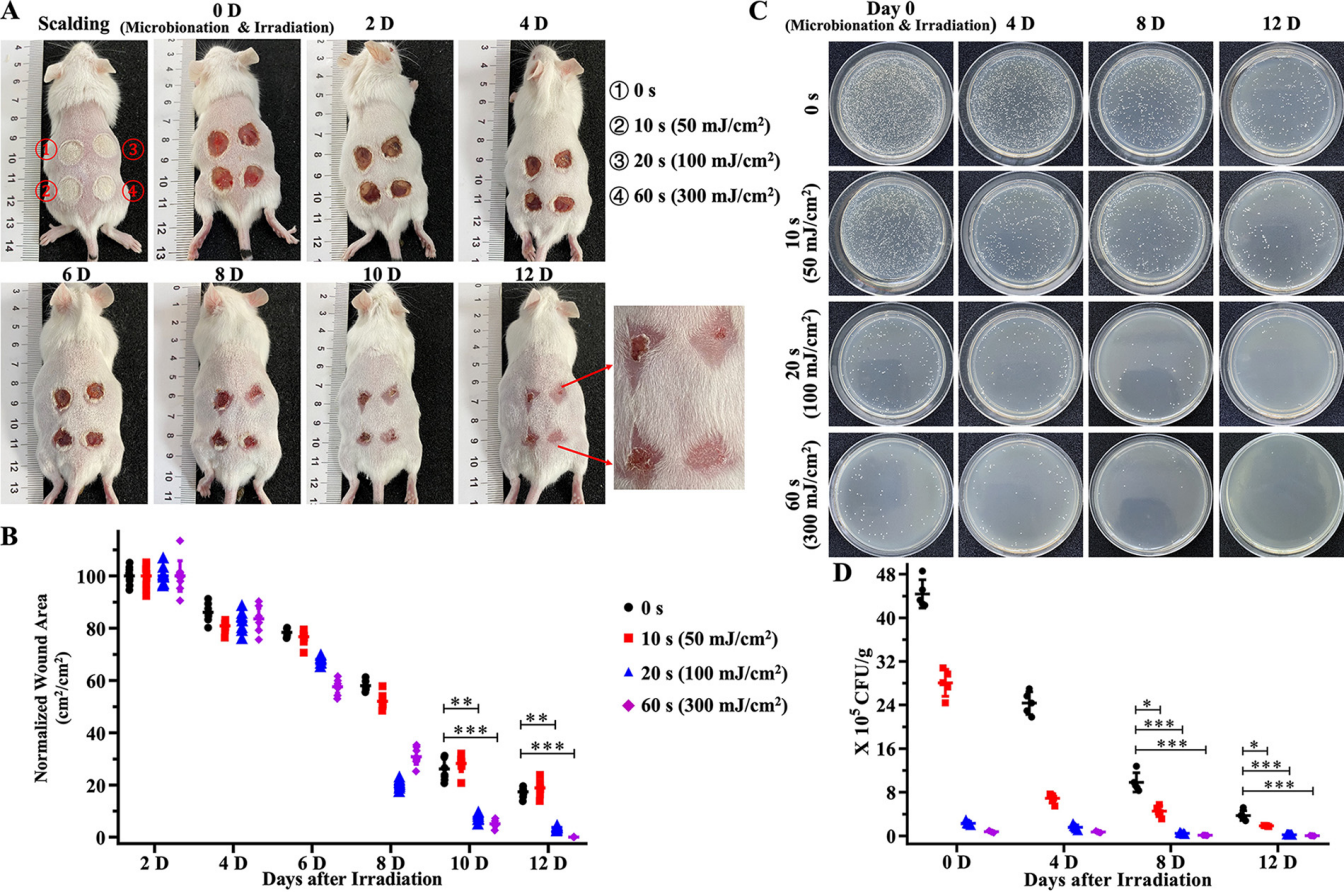
Fungicidal effect and influence on the wound healing time of the 275-nm UVC-LED light on C. albicans inoculated onto the scalded dorsal skin of mice. (A) The infected dorsal skin of the mice was treated with the 275-nm UVC-LED light for 0, 10 s (50 mJ/cm^2^), 20 s (100 mJ/cm^2^), or 60 s (300 mJ/cm^2^) with a power density of ~5 mW/cm^2^. (B) Quantitative analyses of wound area by ImageJ software (*n* = 10). (C and D) Time course fungal counts in C. albicans-infected wounds after treatment with the 275-nm UVC-LED light for different time periods. Skin tissues of different groups were sampled on days 0, 4, 8, and 12. Tissue suspensions were diluted with sterile saline solution and plated in the PDA culture dishes (*n* = 5). Images were taken 18 to 24 h after inoculation. *, *P* < 0.05; **, *P* < 0.001; ***, *P* < 0.001.

As indicated in Fig. 5A and B, the 275-nm UVC-LED light also presented good fungicidal effects in the mouse model of C. albicans-induced SSTIs and shortened the wound healing time. On day 8, both the scabs fell off in the 60 s (30.00% ± 3.83% unhealed) and 20 s (23.06% ± 6.88% unhealed) irradiation groups, with a significant reduction in wound area compared with the groups irradiated for 0 s (58.32% ± 2.08% unhealed) and 10 s (52.16% ± 2.64% unhealed). The wounds in the 60-s irradiation group had completely disappeared on day 12, whereas 5.12% ± 1.77%, 17.72% ± 1.81%, and 18.84% ± 2.78% of unhealed wounds remained observable in the 20-s, 10-s, and 0-s irradiation groups. The recovery period was significantly different between the 275-nm UVC-LED light-treated and nontreated groups. Meanwhile, the colonizing C. albicans organisms in the infected skin and soft tissues were also quantified. As shown in Fig. 5C and D, compared with the 0-s irradiation group, irradiation by the 275-nm UVC-LED light also led to a significant reduction in the colonization by C. albicans in subcutaneous tissues. Only very small amounts of C. albicans organisms could be detected in the skin tissues of the 20-s (1%) and 60-s (0.06%) irradiation groups on day 8. In contrast, there were still more than 10% residual C. albicans organisms in the 10-s irradiation group. On day 12, there were no C. albicans organisms detected in the 60-s irradiation group. The fungal clumps observed in the tissues infected by C. albicans were fewer and smaller (Fig. 5C). Ubiquitous inflammatory cell infiltration, neutrophil infiltration, and necrosis were observed in the skin tissues. On day 12, the wounds of the 60-s irradiation group were completely healed. No C. albicans clumps or inflammatory cells were observed throughout the slice. Instead, significant neovascularization and a large number of newborn hair follicle tissues were ubiquitous, and the skin structure was almost completely intact. In contrast, there were still massive neutrophil infiltration, lymphocytes, and a small number of necrotic tissues observable in the 10-s and 0-s irradiation groups. C. albicans clumps had infiltrated from the surface of the skin into deep muscle tissue. In addition, even though the C. albicans clumps were no longer visible in the 20-s irradiation group, the inflammatory response remained severe (Fig. 4B).

### UVC-LED light at 275 nm induced neither DNA damage nor epidermal lesions in mouse skin.

**FIG 6:**
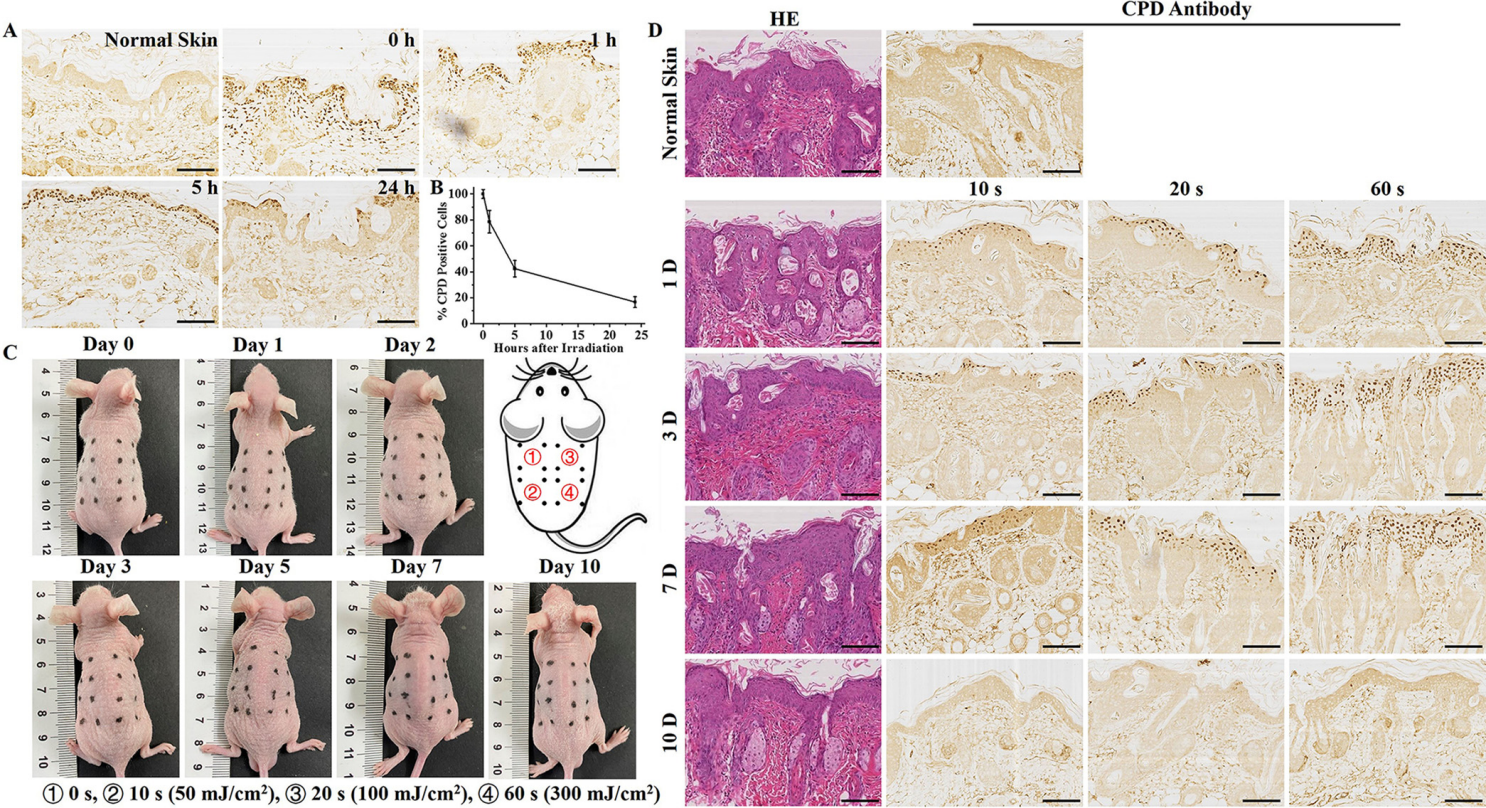
Time course analysis of CPDs following 275-nm UVC-LED light irradiation. (A) Dorsal skin of the mice singly irradiated with the 275-nm UVC-LED light for 60 s (300 mJ/cm^2^) was collected immediately posttreatment as well as 1, 5, and 24 h postirradiation. CPD-expressing cells were detected by immunohistochemistry (magnification, ×200). Scale bar, 200 μm. (B) Percentages of the CPD-expressing cells per visual field (magnification, ×200) were enumerated (*n* = 5). (C and D) Gross appearances of mice and histology analyses of skin samples after irradiation with the 275-nm UVC-LED light for 0, 10 s (50 mJ/cm^2^), 20 s (100 mJ/cm^2^), or 60 s (300 mJ/cm^2^) seven times. Scale bar, 100 μm.

After a single irradiation by the 275-nm UVC-LED light for 60 s, the cyclobutane pyrimidine dimer (CPD)-expressing cells were detectable immediately and distributed mainly in the stratum spinosum. Six hours after irradiation, they were detectable in the upper stratum spinosum. Only tiny CPD-positive cells were detected on the surface of the epidermis 24 h after irradiation (Fig. 6A). In addition, mice quickly repaired the generated CPD with the 275-nm UVC-LED light. Only 41% of the CPD-expressing cells were left 5 h after irradiation and 16% were left 24 h after irradiation (Fig. 6B). The epidermal damage induced by multiple irradiations with the 275-nm UVC-LED light were also investigated. Compared with the nonirradiated mice, neither sunburn nor desquamation was observed in the dorsal skin of the mice irradiated with the 275-nm UVC-LED light, regardless of the irradiation time (Fig. 6C). Histological analysis of the CPD-positive cells further confirmed the safety of the 275-nm UVC-LED light. Seven treatments with irradiation did not lead to a significant accumulation of the CPDs in the epidermis, and no CPD was detectable 3 days after cessation of the irradiation (Fig. 6D). In addition, no tissue damage, necrosis, or inflammation was observed after irradiation by the 275-nm UVC-LED light, and the morphology and structural integrity of the cells did not differ from those of the nonirradiated group.

## DISCUSSION

SSTIs are infectious diseases with high incidence and complex and diverse conditions. The main management for mild SSTIs is the topical use of antibiotics (10, 11). However, the emergence of large numbers of drug-resistant microbes and changes in pathogenic species make the problem of refractory SSTIs increasingly serious and present growing challenges in SSTI management (51). In addition, it is cumbersome to administer topical antibiotics multiple times a day. Some of the odors are unacceptable to patients, resulting in negative therapy of patients and relapse. SSTIs urgently necessitate the development of new alternative therapies.

UV light is currently used in the clinic to treat acute and chronic infections mainly in a photochemical reaction (photodynamic therapy [PDT]), with reasonable success and without significant consequences (52). But direct use of its bactericidal effect for disease therapy is still relatively rare, and the use of UVC irradiation for treatment of wound infections has remained at an early stage until now. The germicidal mechanism of UVC light mainly relies on its DNA-damaging effect caused by a variety of mutagenic and cytotoxic DNA lesions such as CPDs. Different UVC wavelengths have different degrees of sterilization effectiveness and mechanisms. In addition to the germicidal mechanism of UVC, which is mainly related to absorption of the light by nucleic acid components, wavelengths between 200 and 222 nm are also well absorbed by peptide bonds and amino acids (tryptophan and tyrosine) (53). UVC at these wavelengths is expected to be absorbed first by the proteins and/or other biomolecules in the stratum corneum layer before reaching nuclei of the epidermal cells. Therefore, it can only penetrate small organisms, such as bacteria or viruses, and cannot affect larger biological samples, such as mammals. Its antimicrobial effectiveness is relatively lower but safer. However, a light source of 200 to 222 nm with high quality is difficult to obtain and very expensive. The wavelength of ~254 nm is considered to inactivate microbes mainly by directly attacking the nucleic acid structure. It has a much stronger sterilization effect and the light source is very inexpensive. However, it is hazardous to human health, being both carcinogenic and damaging to eye tissues, and can hardly be applied to living beings. Therefore, it is mainly used for environmental disinfection in clinical and daily life (27). The wavelength at 260 to 265 nm is considered to inactivate microbes mainly by nucleic acid damage and CPD damage (41). A wavelength of 270 to 280 nm is considered to inactivate microbes mainly by destroying the protein structure of the microbes and interfering with the function of the proteins. Therefore, this wavelength induces less damage to nucleic acid and has a relatively stronger sterilization effect. It has great potential to be used *in vivo*. In addition, the reactive oxygen species (ROS) generated during UVC irradiation can also induce critical damage for microorganisms (54–56). However, it is difficult to control UVC-LED intensity to such a narrow beam in a realistic system. As described in the light source section, the 275-nm UVC-LED light source emitted radiation in the range of 265 to 285 nm, with a central output wavelength of 275 nm. Therefore, the germicidal mechanism should be the result of the combined damages.

Inspired by the good antimicrobial effect of the 222-nm UVC-LED light reported in the literature and its limitation of the light source (35, 37, 39, 57), we focused on looking for a more easily available light source and safer wavelength. Comprehensively considering the characteristics of different UVC wavelengths, a UVC-LED lamp tube with a central irradiation wavelength of 275 nm (275 ± 10 nm) was self-designed and assembled in this research. In terms of the 275-nm UVC-LED lamp tube itself, in contrast to the 222-nm wavelength, the 275-nm UVC-LED light source has been made commercially available. In addition, it is a relatively inexpensive and high-quality light source which can be accessed from the market (40). Therefore, it is convenient for the design and mass production of the lamp tubes. In terms of antimicrobial effectiveness, it was demonstrated in this study that the 275-nm UVC-LED light could inactivate both bacteria and fungi with very short irradiation time *in vitro*, indicating its relatively stronger sterilization effect. In addition, in mouse models of SSTIs caused either by MRSA or by C. albicans, the 275-nm UVC-LED light could shorten the healing time of wounds. By measuring the microbes loaded in the wound tissues, it was determined that the wound healing time was shortened mainly owing to the significant antimicrobial effects of the 275-nm UVC-LED light. Irradiation using the 275-nm UVC-LED light for 60 s could inactivate more than 95% of the microbes attached to the wound area, so as to inhibit wound area microbial infection and shorten the wound healing time. In addition, the treatment is also very simple and easy to administer, with no noise and bad smell, and does not cause discomfort to patients and medical staff. Patients can even take the light home and operate it autonomously. Therefore, it is a promising physical therapy for skin infections. We expect that it could be applied easily and quickly to other scenarios, such as for infections of wound sites after surgery, diabetic foot, bedsores, etc. Moreover, by designing the flexibility of the 275-nm UVC-LED lamp tube, we are also considering the possibility of its application in deep infections *in vivo* (Helicobacter pylori infection, lung infection, cholecystitis, etc.).

Considering the effectiveness, safety, and portability of the 275-nm UVC-LED light, it is a promising physical therapy for skin infections and a potential method to promote the healing of infected wounds. However, there are still many factors to be considered before large-scale clinical application. In this study, although the 275-nm UVC-LED light had a good *in vivo* therapeutic effect, the mouse models involved superficial infections. Primarily limited by the tissue penetration depth of the 275-nm UVC-LED light, its specific application scenarios need further study. In addition, the mechanism of the UVC light for antimicrobial effect and shortening wound healing time was widely regarded as inducing DNA or protein damage in pathogens and thus inhibiting the proliferation of pathogens. Whether there are other mechanisms, such as inducing an enhanced immune response to promote antimicrobial effect, inducing the increased expression of cold-inducible RNA binding protein (58, 59), or thermal effect, remains to be studied. Due to the diversity of pathogens, the differences in the lesion location, and the depth of infections, the relationship of the irradiance of the 275-nm UVC-LED light to SSTIs caused by different microbes still needs to be explored. Meanwhile, there is also research on the photorepair ability of some organisms which have the ability to recover after irradiation with UV and become infective again (60). Spores of four *Frankia* strains were exposed to short-wavelength UVC radiation (254 nm) for 10 min, followed by reactivation with visible white light for the same period of time. As revealed by scanning electron microscopy, all the tested strains showed repairing activity induced by white light with increased spore germination and protein content. The normal shape of *Frankia* hyphae was almost restored, too (61). However, the irradiation dose was relatively low. There are also some UVC-resistant bacteria, like Listeria monocytogenes (62), Antarctic bacteria (63), and Bacillus subtilis (25), being reported. Therefore, this is a factor that must be considered before this technology can be confidently used on a larger scale.

Meanwhile, the DNA and epidermal damage induced by single and multiple irradiations with the 275-nm UVC-LED light were also investigated. It was demonstrated in this study that the 275-nm wavelength is relatively safe in skin application, showing neither DNA damage nor epidermal lesions in mouse skin with 60-s irradiation. However, although the 275-nm UVC-LED light is relatively safe for skin with 60-s irradiation, it should be noted that the influence associated with immediate and long-term irradiation with other irradiation dosages is still unknown. On one hand, the CPDs induced by the 275-nm UVC-LED light irradiation are the most predominant and persistent type of DNA lesion, and they have great influence on the recovery of dermal fibroblasts. Decrease in the repair rates of the lesions in dermal fibroblasts may cause severe structural distortions in the DNA molecule, affecting important cellular processes such as DNA replication and transcription and ultimately leading to mutagenesis and tumorigenesis (57). In this study, the generation and repair of CPDs were monitored for only 7 days; the long-term generation and repair of CPDs with other irradiation dosages are still unknown, and the long-term influence on skin is also unknown. On the other hand, the thermal effect caused by long-time and high-dose irradiation is also one of the factors to be considered. In addition, there are also reports about the ROS generated by UVC irradiation, which can induce the critical damage (54). Furthermore, the generated ROS can induce multiple failure in cell components such as protein denaturation, DNA oxidation, and lipid oxidation (64, 65).The long-term influence of ROS generated by UVC irradiation should also be considered. Therefore, there are still many safety considerations of 275-nm UVC-LED light to be explored before clinical applications.

To summarize, the results in this study demonstrated that the 275-nm UVC-LED light could inactivate both bacteria and fungi with very a short irradiation time *in vitro* and induced neither DNA damage nor epidermal lesions in mouse skin. It shortened the wound healing time on scalding combined with SSTIs induced by either MRSA or C. albicans *in vivo* through its significant antimicrobial effect. Thus, the 275-nm UVC-LED light is a promising physical therapy candidate in the battle against multiresistant bacterium- and fungus-induced SSTIs.

## MATERIALS AND METHODS

### Materials.

Luria-Bertani (LB) medium, Luria-Bertani agar (LBA), potato dextrose broth (PDB), and potato dextrose agar (PDA) were obtained from Solarbio Life Science. Cyclobutane pyrimidine dimer (CPD) antibody was purchased from Kamiya Biomedical Company (USA). Enterococcus faecalis ATCC 29212, Escherichia coli ATCC 25922, Klebsiella pneumoniae ATCC 700603, methicillin-resistant Staphylococcus aureus (MRSA; 2104270609, clinical isolate), Pseudomonas aeruginosa ATCC 27853, Candida krusei ATCC 6258, Candida albicans ATCC 90028, and Candida glabrata ATCC MYA-2950 were obtained from the First Affiliated Hospital of the Xi’an Jiaotong University.

### UVC-LED light source.

Figure 1A and B show the appearance of the disinfection lamp tube, consisting of a light tube, a constant-current-source drive circuit, and a 3.7-V lithium battery. A protective tube made of a heat-shrinkable polymer was installed to waterproof the equipment. The total length of the light tube was ~29 cm, with a width of 2 mm. A total of 15 275-nm UVC 5050 LED chips (PCD-10-V1; Photon Wave Co., Ltd., South Korea) were mounted along the slim printed circuit board with a gap distance of 2 cm. The actual deep UV emission area for each LED chip was 550 μm by 550 μm. During the working period, 100 mA of DC current flowed through each chip, and the average light output power of the LED chips was measured as 12 mW. The viewing angle from each LED was 125 degrees. The irradiance at different distances from the LED chip was measured by an LS125 multiprobe UV irradiation meter (LinshangTec, Shenzhen, China). The distance between the lamp tube and the irradiation meter was controlled using an electronic universal testing machine (UTM6202; Shenzhen Suns Technology Stock Co., Ltd.) As shown in Fig. 1C, the UVC-LED lamp tube emitted radiation in the range of 265 to 285 nm and the central output wavelength was 275 nm. As shown in Fig. 1D, the irradiance at 1 mm was ~8.2 mW/cm^2^ and it decreased exponentially moving away from the light source. It was ~5 mW/cm^2^ at 3 mm and ~1.5 mW/cm^2^ at 5 mm from the light source.

### Bacteria and fungi irradiated by the 275-nm UVC-LED light *in vitro*.

The concentrations of the bacteria and fungi used in this study were calculated from the absorbance value at 600 nm using the standard curves in Table S1 in the supplemental material. For experiment 1, as shown in Fig. S1, 50 μL of 1 × 10^6^ CFU/mL of bacteria or fungi was inoculated into standard 90-mm LBA or PDA culture dishes. Then, the culture dishes were irradiated directly by the UVC-LED lamp tube for 0 s, 5 s (7.5 mJ/cm^2^), 10 s (15 mJ/cm^2^), 20 s (30 mJ/cm^2^), or 60 s (90 mJ/cm^2^) (*n* = 3). Black lines were drawn on the bottom of the culture dishes to indicate the position where we put the lamp tube. The distance between the lamp tube and agar surface was ~5 mm, and the irradiance of the UVC-LED light was ~1.5 mW/cm^2^ (dose = irradiance × irradiation time). The culture dishes were then cultured at 37°C. For experiment 2, as shown in Fig. S2, self-designed three-dimensionally (3D) printed culture plates (length by width by height = 125 by 85 by 9 mm; well depth, 2 mm; well diameter, 8 mm; well space, 20 mm), made of high-temperature resistant resin, were used (Keheng Hand Model Co., Ltd., China). Thirty-microliter volumes of 1 × 10^6^ CFU/mL of microbial strains were sucked into the self-designed culture plates. The culture plates were irradiated by the UVC-LED lamp tube for 0 s, 5 s (25 mJ/cm^2^), 10 s (50 mJ/cm^2^), 20 s (100 mJ/cm^2^), or 60 s (300 mJ/cm^2^) (*n* = 3). The distance between the lamp tube and liquid surface was ~3 mm, and the irradiance of the UVC-LED light was ~5.0 mW/cm^2^. After irradiation, 5 μL of the sample was dropped into 96-well culture plates to which 95 μL of fresh culture medium had been added. The mixtures were cultured at 37°C and 200 rpm for 12 h, and the absorbance at 600 nm was recorded by a Varioskan Flash multilabel reader (Thermo Fisher). The CFU of bacteria or fungi were calculated based on the standard curves of the absorbance at 600 nm to the concentrations (in CFU per milliliter) of the different bacterial or fungal strains. Viability of bacteria and fungi was calculated using this formula: survival rate = mean CFU of the irradiated group/mean CFU of the nonirradiated group. Another 5 μL of the irradiated microbial solution was mixed with 25 μL of the sterile saline solution, and the mixture was inoculated into culture dishes and cultured at 37°C. Photos of the culture dishes in experiments 1 and 2 were taken after 18 to 24 h for bacteria and after 36 to 40 h for fungi with an iPhone 11.

### Animals.

Normal male BALB/c nude mice (~20 g; 6 to 7 weeks) and Kunming mice (~25 g; 5 to 6 weeks) were used in this study. All animals were supplied by the Medical Animal Test Center of Xi’an Jiaotong University, and the study protocols were approved by the Institutional Animal Care and Use Committee of the Ethics Committee of Health Science Center, Xi’an Jiaotong University (ethical approval code 2017-609).

### Effect of irradiation by the 275-nm UVC-LED light on the epidermis of mouse skin.

Normal male BALB/c nude mice (~20 g; 6 to 7 weeks) were used. Five mice were anesthetized by 4% chloral hydrate and four ~1-cm squares were marked on the dorsal skin. The marked areas were subsequently subjected to single irradiation by the 275-nm UVC-LED light for 60 s (irradiance, ~5 mW/cm^2^; dose, ~300 mJ/cm^2^). Skin samples within the square were collected along the sides of the square at 0, 1, 5, and 24 h after irradiation and soaked in 4% paraformaldehyde solution (*n* = 3 for each time point).

Ten male BALB/c nude mice (~20 g; 6 to 7 weeks) were anesthetized by 4% chloral hydrate and four ~1-cm squares were marked on the dorsal skin. The marked areas were subsequently subjected to daily irradiation by the 275-nm UVC-LED light for 0 s, 10 s (50 mJ/cm^2^), 20 s (100 mJ/cm^2^), or 60 s (300 mJ/cm^2^) for 7 days (total irradiation, seven times for each area). The irradiance was ~5 mW/cm^2^. Gross appearances of irradiated skin were observed immediately after irradiation, and the dorsal skin of the irradiated areas was sampled on days 1, 3, 7, and 10 (3 days after cessation of irradiation). Skin samples were soaked in 4% paraformaldehyde solution for immunohistochemistry analysis. The immunohistochemistry analysis in this study was carried out by Servicebio (Wuhan, China), and detailed processes are described in the supplemental material.

### *In vivo* anti-SSTI assay.

Forty normal male Kunming mice (5 to 6 weeks) weighing ~25 g were anesthetized by isoflurane using an anesthesia machine (R640; RWD Life Science, China). After skin preparation and disinfection, a circular iron (diameter, 8 mm) was used to burn the dorsal skin (300°C, 10 s). Mice were injected subcutaneously with 5 mg/kg (of body weight) of meloxicam (Aladdin, China) for 4 days (once per day) after scalding for pain relief. Two days after scalding, entire skin tissues and small amounts of adhesive muscle tissues of the scalded area were removed carefully following the circular edge of the scalded area. Thirty microliters of 1 × 10^7^ CFU/mL of MRSA or C. albicans dispersed in saline solution was applied evenly onto the surface of each wound. After the microbial fluid was completely absorbed (1.5 to 2 h) (57, 66), 275-nm UVC-LED light irradiation was performed for 0, 10, 20, or 60 s. As shown in Fig. S3, the lamp bead was placed in the center of the round wound as much as possible. Mice were put back in the original feeding environment with two mice per cage. Gross appearances of the wound were observed every 2 days, and the area of the wound was measured using ImageJ software. The average wound area on day 2 was used as the reference (100% unhealed) to normalize the wound area on other days. The wound area on days 4, 6, 8, and 12 (after irradiation with 275-nm UVC-LED light for 0 s, 10 s, 20 s, or 60 s) was normalized separately compared with the corresponding average wound area on day 2. Meanwhile, the entire skin tissues of the wound area were removed at 0, 4, 8, and 12 days after irradiation (*n* = 5 for each time point), and part of the skin tissues were weighted in a sterile environment and soaked in an appropriate amount of sterile saline solution (adding 100 μL of sterile saline solution into 1 mg of skin tissues). Tissues solutions were obtained using a tissue lyser (Tissuelyser LT; Servicebio) and plated into culture dishes. After culturing overnight, pictures of the culture dishes were taken with an iPhone 11. The colonies were counted using the ImageJ software or by eye. In addition, on day 0 and day 12 after irradiation, skin tissues of the wound area were collected (*n* = 3 for each time point) and soaked in 4% paraformaldehyde solution for hematoxylin-eosin (H&E) staining.

### Statistical analysis.

The data were expressed as means ± standard deviations (SD). The statistical difference between the two groups was determined using Student’s *t* test (GraphPad Prism 8.0). *P* values less than 0.05 were considered statistically significant.
