# Supplementary material for: UV C Light from a Light-Emitting Diode at 275 Nanometers Shortens Wound Healing Time in Bacterium- and Fungus-Infected Skin in Mice
Source: Microbiol Spectr. 2022 Dec 1;10(6):e03424-22. doi: 10.1128/spectrum.03424-22 (PMC9769979; doi:10.1128/spectrum.03424-22)
Supplement: Supplemental file 1 — Supplemental text, Fig. S1 to S3, and Table S1. Download spectrum.03424-22-s0001.pdf, PDF file, 0.5 MB [file spectrum.03424-22-s0001.pdf]

Supplementary Material

1.1 Histological and Immunohistochemical Analysis

Skin tissues were soaked in 4% paraformaldehyde solution and fixed for more than 24 hours, dehydrated and embedded with paraffin. Then, 4 μm-thick tissue sections were prepared. After deparaffinization and rehydration, antigen retrieval of tissue sections was performed by incubating the sections in citric acid antigen repair buffer (G1202, Servicebio, China). After that, 3% hydrogen peroxide was used to block endogenous peroxidase activity and 3% bovine serum albumin (BSA) was used for serum sealing. To detect the formation of CPD, the sections were then incubated with anti-CPD monoclonal antibodies (MC-062, Kamiya Biomedical Company, USA) overnight at 4 °C. Then, the sections were placed in Phosphate Buffer Saline (PH 7.4) and washed three times. After the sections were slightly shaken and dried, the tissues were covered with secondary antibodies (horseradish peroxidase (HRP) labeled). After rinsing three times in PBS for 5 min each, the color reaction was developed by the addition of diaminobenzidine. CPD-positive cells were quantified by counting the cells in 5 random visual fields of each section (200× magnification).

1.2 Supplementary Figures

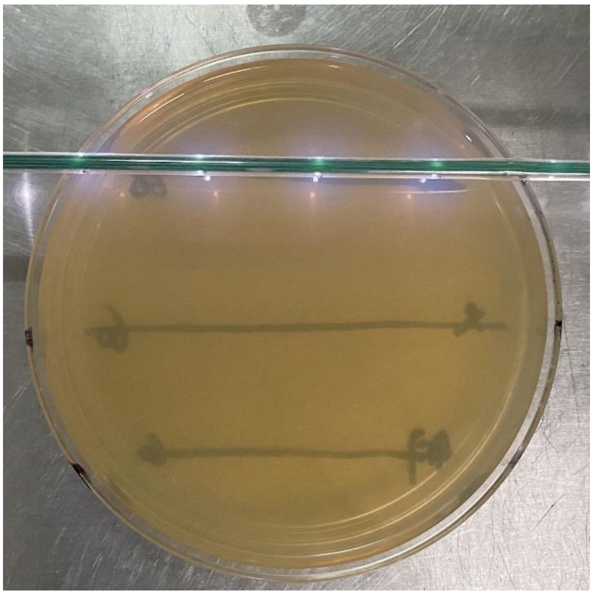

Supplementary Figure 1. Bacteria or fungi inoculated in culture dishes were directly irradiated by the 275-nm UVC-LED light with the power density of ~ 1.5 mW/cm^2.

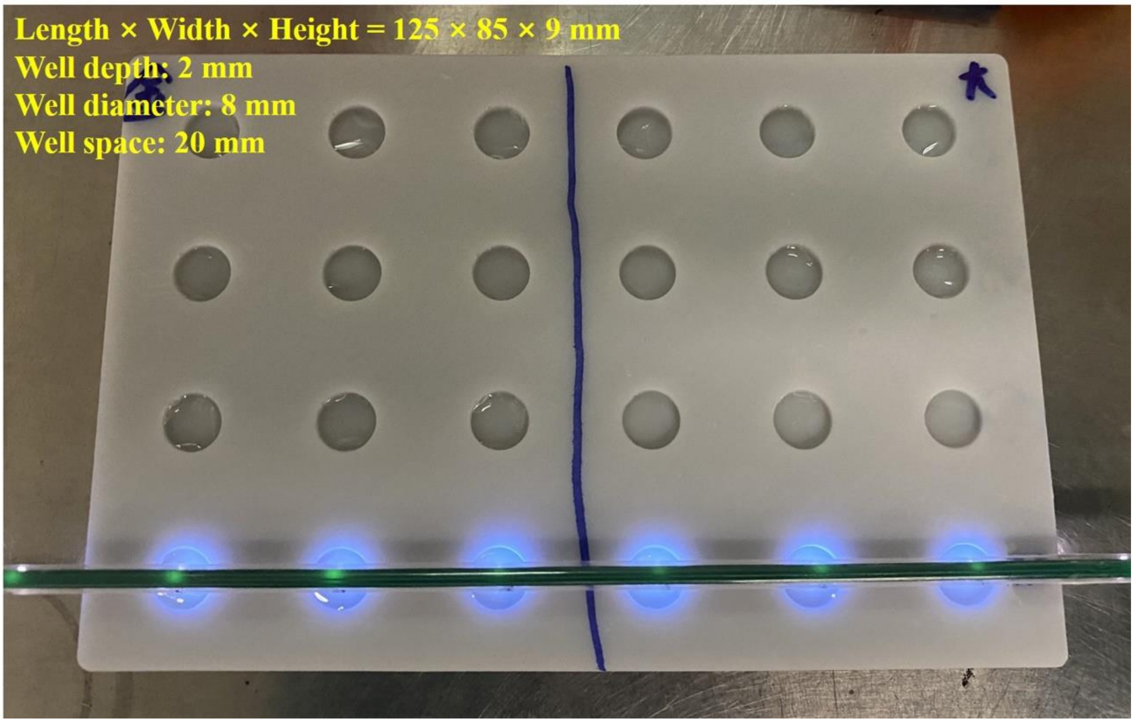

Supplementary Figure 2. Bacteria or fungi were irradiated by the 275-nm UV-LED light in saline solution with the power density of ~ 5 mW/cm^2 in self-designed culture plates.

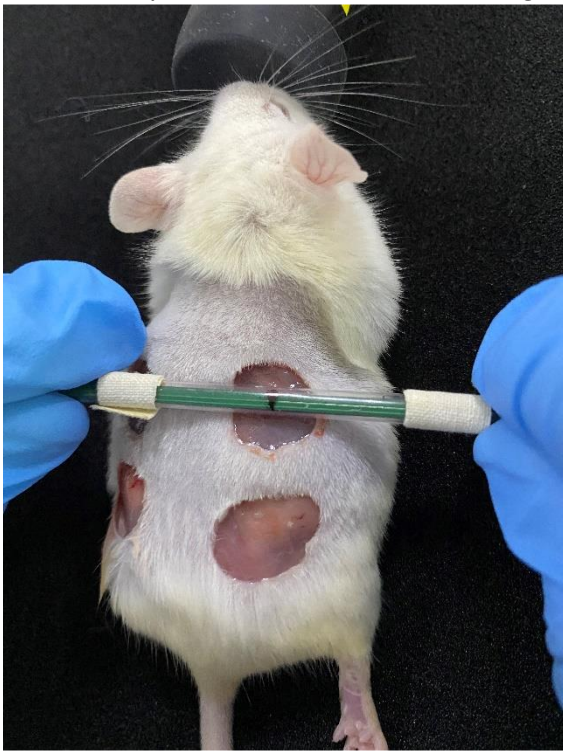

Supplementary Figure 3. Relative position of the 275-nm UVC-LED lamp tube and the round wound in anti-SSTIs Assay. The lamp bead was placed in the center of the round wound as much as possible.

1.3 Standard Curves of Different Bacteria and Fungi

These standard curves were obtained by the following process. 1. The bacterial or fungal

solution was diluted for different multiples (1, 1/2, 1/5, 1/10, and 1/50. n = 5.). 2. The absorbance at 600 nm was measured by the Multilabel Reader VARIOSKAN FLASH (Thermo Fisher, Volume = 100 µL). 3. The diluted bacterial or fungal solution were diluted again (1/100-1/1000000) and inoculated in standard 90-mm culture dishes (Volume = 20 µL). 4. The number of colonies was counted after culturing overnight. 5. Data analysis and draw the standard curves. ΔAbs_{600nm} = Absorbance of the bacterial or fungal solution - Absorbance of the LB or PDB culture medium.

The standard curves had been tested and verified by our lab. They are suitable for calculating the concentrations of our microbial strains and culture conditions. However, the standard curves might be different in different labs, microbial strains and culture conditions.

Supplementary Table 1. Standard Curves of Different Bacteria and Fungi

Name	Standard Curves（X 10^9 CFU/mL）
Methicillin-Resistant *Staphylococcus aureus*	$(\Delta Abs_{600nm}-0.0215)/0.1068$
Escherichia coli	$(\Delta Abs_{600nm}-0.017)/1.2309$
Pseudomonas aeruginosa	$(\Delta Abs_{600nm}+0.0081)/0.1375$
K. pneumoniae	$(\Delta Abs_{600nm}-0.0066)/0.0892$
Enterococcus faecalis	$\Delta Abs_{600nm}/0.1688$
Candida albicans	$(\Delta Abs_{600nm}+0.0008)/9.63$
Candida krusei	$(\Delta Abs_{600nm}+0.0012)/8.2314$
Candida glabrata	$(\Delta Abs_{600nm}-0.0043)/3.0688$
